# Supplementary material for: Purely self-rectifying memristor-based passive crossbar array for artificial neural network accelerators
Source: Nat Commun. 2024 Jan 2;15:129. doi: 10.1038/s41467-023-44620-1 (PMC10761713; doi:10.1038/s41467-023-44620-1)
Supplement: Supplementary file 1 — Supplementary Information [file 41467_2023_44620_MOESM1_ESM.pdf]

Supplementary Information

Purely Self-rectifying Memristor-based Passive Crossbar Array for Artificial Neural Network Accelerators

Kanghyeok Jeon,† Jin Joo Ryu,† Seongil Im, Hyun Kyu Seo, Taeyong Eom, Hyunsu Ju, Min Kyu Yang*, Doo Seok Jeong*, and Gun Hwan Kim**

Supplementary Information 1

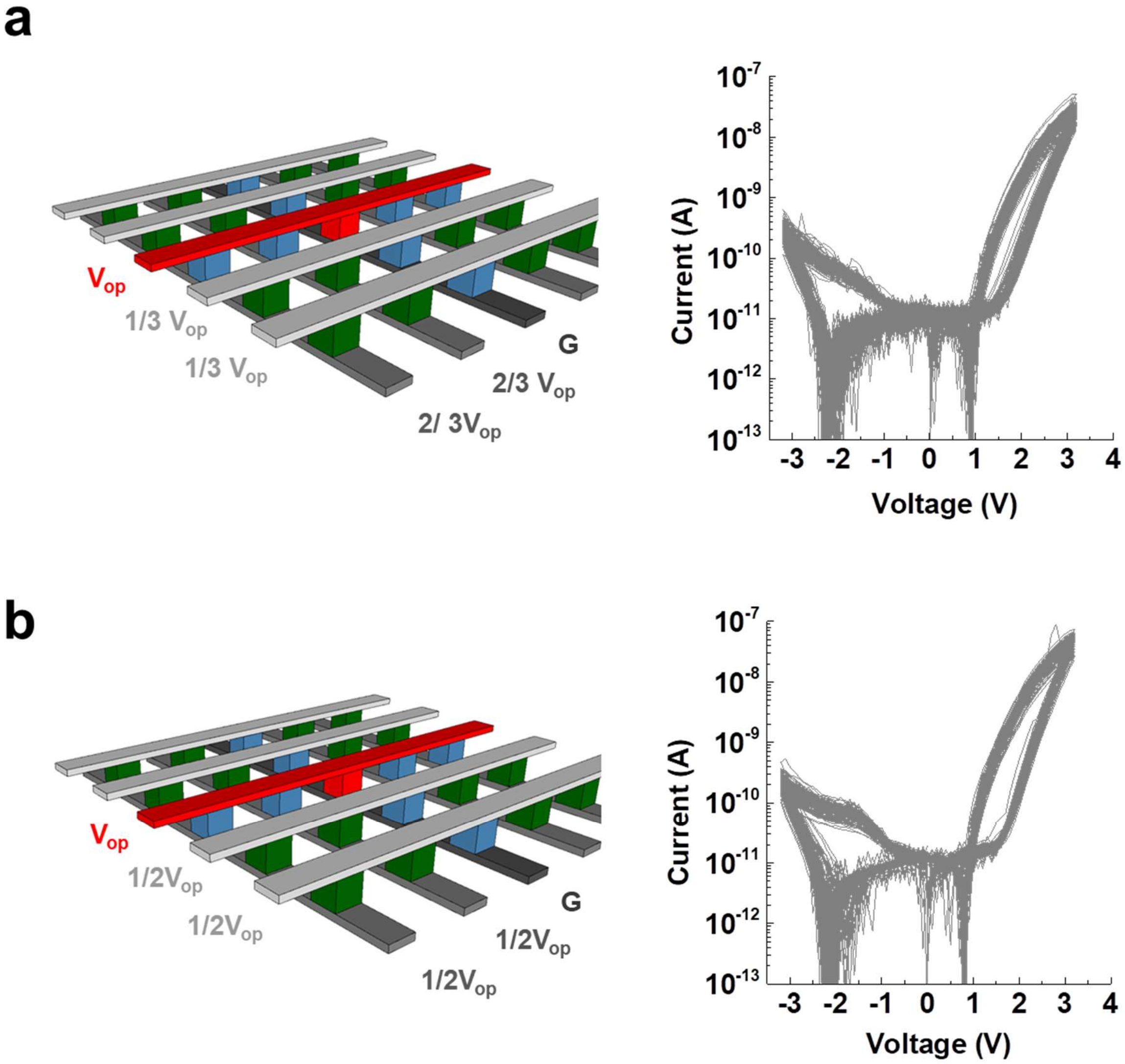

Supplementary Fig. S1: The DC *I-V* behavior of 1 kb CA under different biasing schemes. To investigate the influence of biasing schemes for 1 kb CA, two different bias schemes were applied. a The schematic figure of 1 kb CA utilizing a one-third biasing scheme (1/3 V_{op}) and the measured DC *I-V* characteristics of 1024 cells in 1 kb CA were demonstrated. b Similarly, the schematic figure and DC *I-V* characteristic of 1 kb CA utilizing half biasing scheme (1/2 V_{op}) were depicted. The measured DC *I-V* characteristics under both biasing schemes revealed nearly identical operational currents, approximately 40 pA at 3.2 V and 400 pA at -3.2 V, as well as off-leakage currents at a level of around 10 pA. The minimal difference in operation

between the two biasing schemes can be attributed to the high selectivity and exceptionally low operational and off-leakage currents exhibited by the integrated SRM devices. [1]

Supplementary Information 2

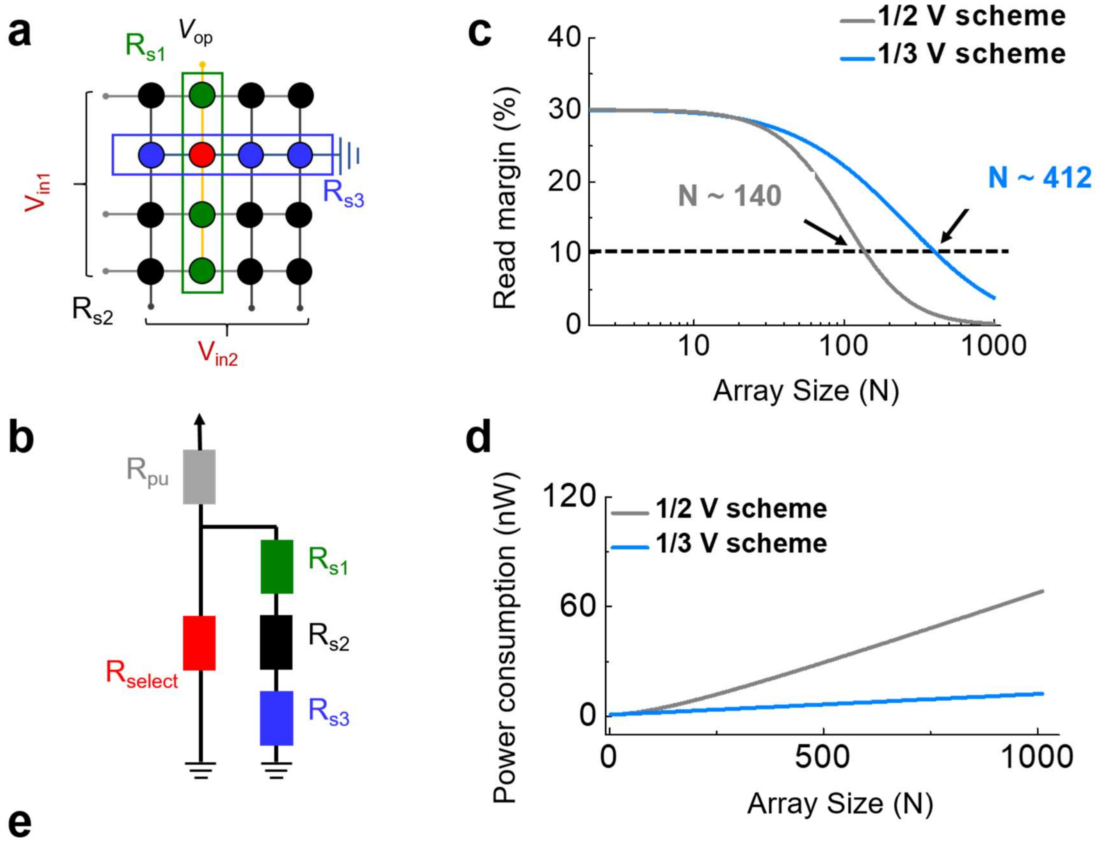

e

	R_{HRS} (Ω)	R_{LRS} (Ω)	R_{s1} (Ω)	R_{s2} (Ω)	R_{s3} (Ω)	R_{pu} (Ω)	N
**1/2 scheme**	4.00E+09	1.00E+09	2.50E+10	1.00E+13	2.50E+10	1.00E+09	32
**1/3 scheme**	4.00E+09	1.00E+09	1.75E+11	4.67E+12	1.75E+11	1.00E+09	32

Supplementary Fig. S2: The numerical analyses of 1 kb CA under different biasing schemes. To investigate the influence of biasing schemes for 1 kb CA, two different bias schemes were applied. a The schematic figure of 1 kb CA under biasing schemes. Under the biasing schemes, the selected cell is connected the bit line biased to V_{op} and word line grounded. However, the unselected cells are partitioned into three groups, which are referred as Rs1, Rs2, and Rs3. The group of Rs1 (green colored cells) are connected bit line biased to V_{op} and word lined biased to inhibit voltage 1 (V_{in1}). The group Rs2 (black colored cells) are connected bit line biased to inhibit voltage 2 (V_{in2}) and word lined biased V_{in1}. The group Rs3 (blue colored cells) are

connected bit line biased to V_{in2} and word lined grounded. b The equivalent circuit of the CA under biasing schemes. c The calculation results of read margin according to each biasing scheme. d The calculation results of the additional power consumption stemming from the unselected cells under each biasing scheme. e The parameters of proposed CA for numerical calculations.

To calculate the read margin under each biasing scheme, the equations (1) to (3) are utilized.

$$\text{Read margin}(\%) = \frac{R_{pu}}{R_{LRS,Read}+R_{pu}} - \frac{R_{pu}}{R_{HRS,Read}+R_{pu}} \tag{1}$$

$$R_{HRS,Read} = \frac{R_{HRS}\times(\frac{R_{S1}}{N-1}+\frac{R_{S2}}{(N-1)^2}+\frac{R_{S3}}{N-1})}{R_{HRS}+(\frac{R_{S1}}{N-1}+\frac{R_{S2}}{(N-1)^2}+\frac{R_{S3}}{N-1})} \tag{2}$$

$$R_{HRS,Read} = \frac{R_{HRS}\times(\frac{R_{S1}}{N-1}+\frac{R_{S2}}{(N-1)^2}+\frac{R_{S3}}{N-1})}{R_{HRS}+(\frac{R_{S1}}{N-1}+\frac{R_{S2}}{(N-1)^2}+\frac{R_{S3}}{N-1})} \tag{3}$$

Supplementary Information 3

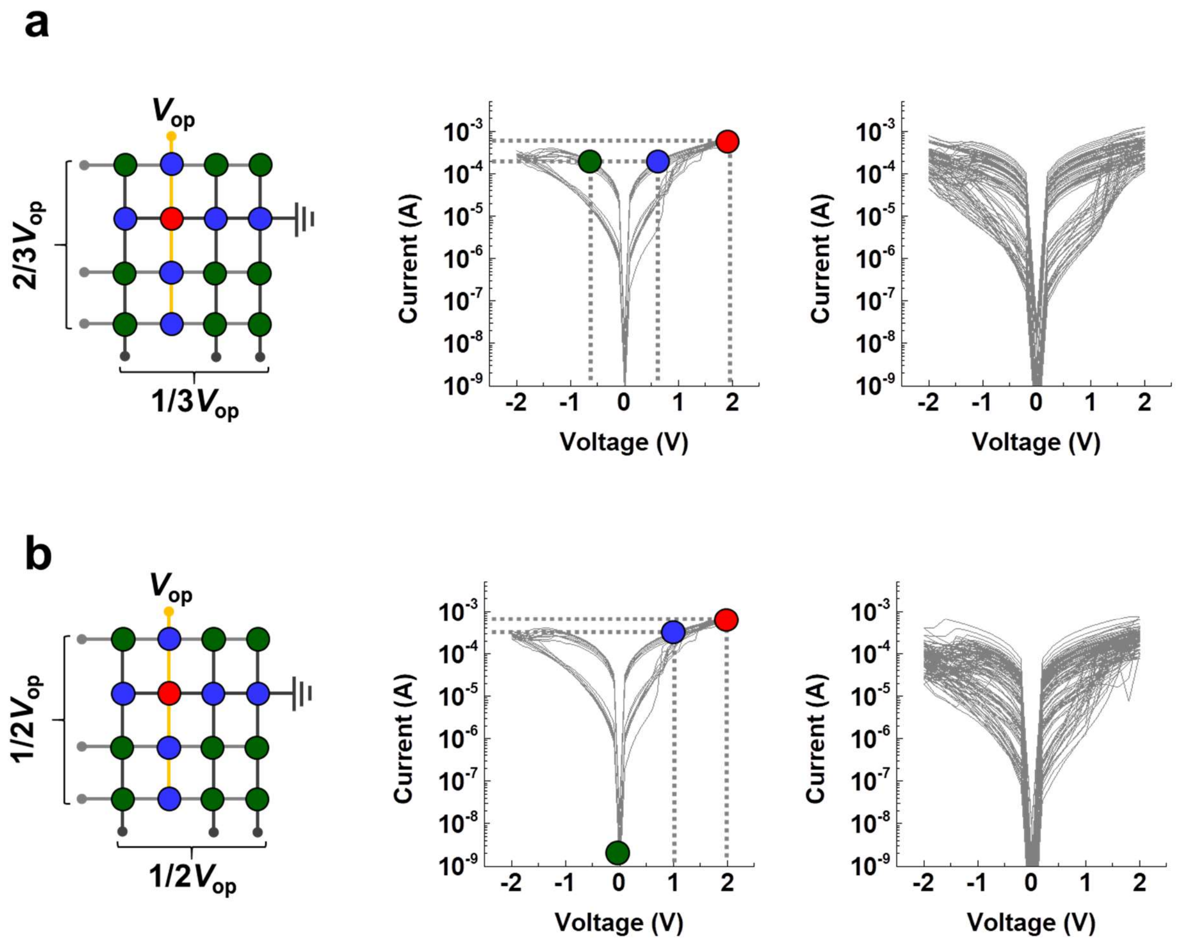

Supplementary Fig. S3: Investigation of biasing schemes on 8 × 8 CA integrated with memristor without selection functionality

The DC *I-V* characteristics of 8 × 8 CAs integrated with memristors without selection functionality were analyzed using different biasing schemes. **a** The schematic diagram of the one-third biasing scheme and the corresponding DC *I-V* characteristic of a single memristor with the resistance states are shown. The DC *I-V* characteristic of the 8 × 8 CA utilizing the one-third biasing scheme is also displayed. Similarly, **b** the schematic diagram of the half-biasing scheme, the DC *I-V* characteristic of a single memristor with the resistance states, and the DC *I-V* characteristic of the 8 × 8 CA with the half-biasing scheme are presented. The CA utilizing the one-third biasing scheme exhibited a relatively lower operational dispersion

compared to the half biasing scheme. However, both cases showed high operational variability, indicating significant interferences between the cells within the CA. This variability can be attributed to the absence of selection functionality in the integrated memristors.

Supplementary Information 4

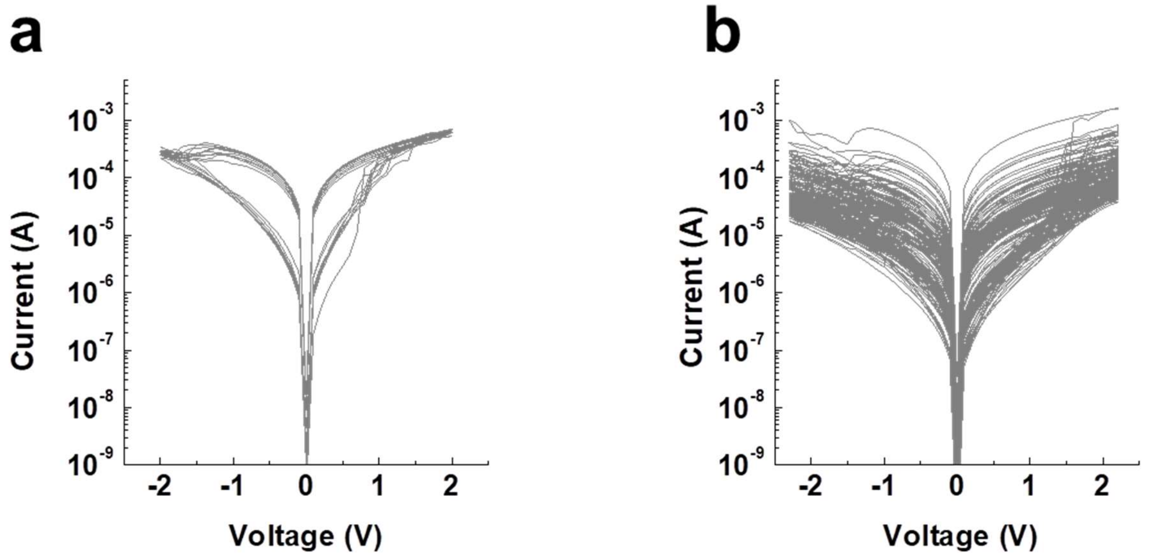

Supplementary Fig. S4: DC *I-V* characteristic of 1 kb CA integrated with the memristor without selection functionality.

To investigate the impact of integrating memristors without selection functionality into higher-density CA, we incorporated memristors without selection functionality into a 1 kb CA and measured the corresponding DC *I-V* curve. **a** The DC *I-V* curve of a single memristor device. **b** The DC *I-V* curve of the 1 kb CA. By comparing the DC *I-V* characteristics of the 1 kb CA with those of an 8×8 CA, as depicted in Figure 6b, it is evident that the DC *I-V* curve of the 1 kb CA exhibits a significantly higher degree of variability. This result indicates that integrating memristors without selection functionality into a higher-density CA has a more pronounced impact on operational variability. Furthermore, this variability becomes more prominent as the density of the CA increases.

Supplementary Information 5

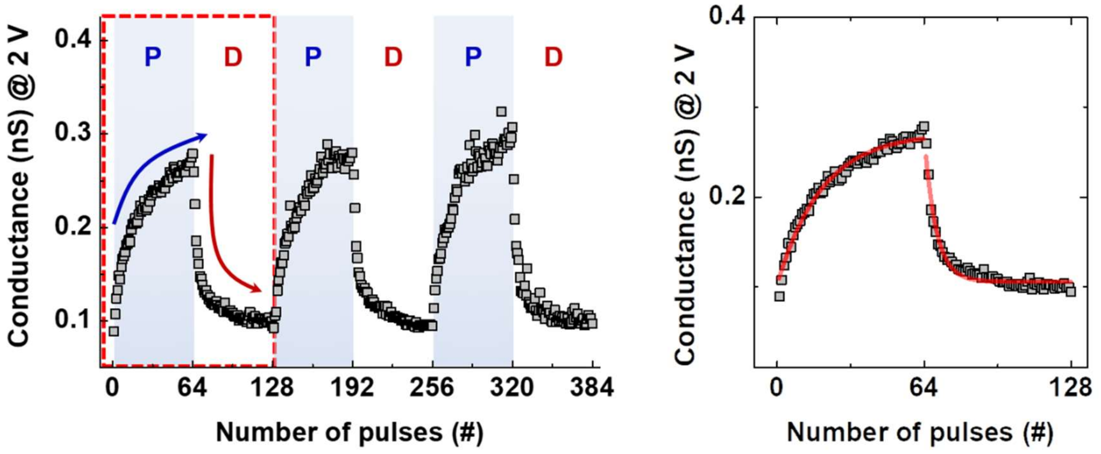

Pulse conditions			Linearity factor		Symmetry factor
Width	Potentiation	Depression	Potentiation	Depression	1.15
200 μs	3.7 V	-3.5 V	0.0514	0.195	

Supplementary Fig. S5: Synaptic properties measured utilizing the SRM-based 1 kb passive CA.

To investigate the gradual resistance change under identical pulse trains, we conducted the ‘potentiation’ and ‘depression’ characteristics which are the synaptic plasticity utilizing the constant step pulse program (CSPP) method. The electrical pulse conditions induced are set to 200 us of width, 3.7 V of potentiation (SET) amplitude, and -3.5 V of depression (RESET) am plitude. Furthermore, the 64-level of pulse number for each potentiation and depression proces s is induced, which is the optimized number of pulses that can exhibit a nearly ideal symmetri c factor for the process. To calculate the linearity factor, we utilized the equation (1)

$$G = \lambda \exp(\alpha x + \beta) + \gamma \quad (1)$$

Where G is the conductance, x is input pulse number, α is a nonlinearity factor, λ, β, and γ are other related coeffcient to determine the fitting. [2]

And, the equation (2) is utilized to calculate the symmetric factor of the operation.

$$\text{Symmetric factor} = \max\left(\frac{\Delta G_D}{\Delta G_P}, \frac{\Delta G_P}{\Delta G_D}\right) \qquad (2)$$

Whrer ΔG_P and ΔG_D are the overall conductance changes in the potentiation and depression processes, respectively, and the max function is used [3]. As a result, the linearity factors of 0.0514 and 0.195 for potentiation and depression, respectively, are calculated. Furthermore, the symmetric factor of these processes is calculated for 1.15.

Supplementary Information 6

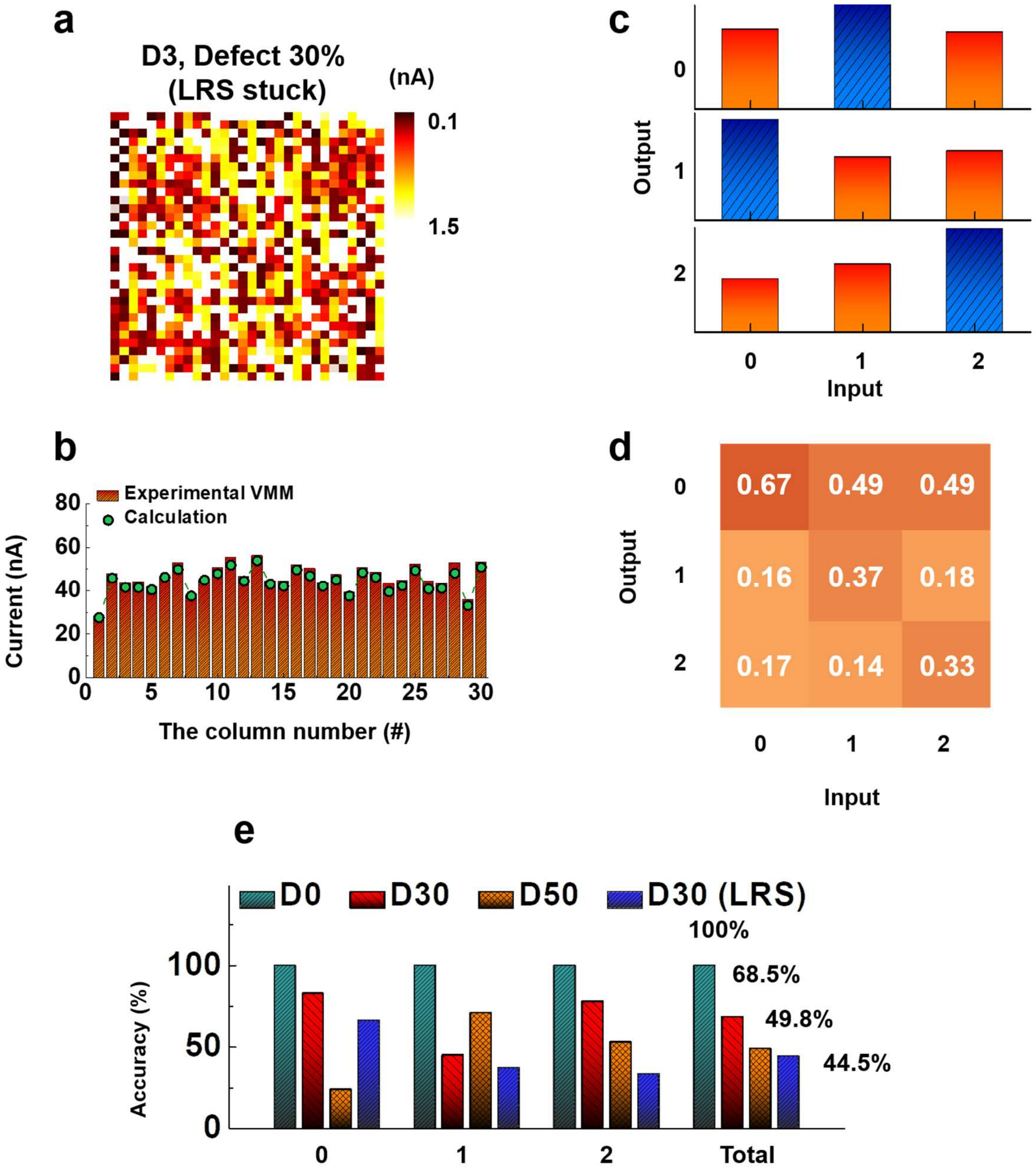

Supplementary Fig. S6: Fully hardware-based demonstration of a single-layer neural network for MNIST data classification utilizing LRS stuck defect rate of 30% (D30, LRS). **a** Trained weight-mapping results of the D30 (LRS) CA. After the training process, all cells in the CA were read out at a 2 V reading voltage. **b** VMM operation results of the D30 (LRS) CA .VMM operation results (red bars), trained weight summations (green circles) are compred to demonstrate the discrepancy between the VMM operation results and calculated values which

indicates the feasibility of the VMM operation. **c** The representative experimental demonstration of the fully hardware-based classification of the MNIST data using the D30 (LRS) CA. The classification result is indicated by the blue bars, which represent the maximum values of the sensed signals. **d** Classification accuracy for each digit based on 6 classification. **e** Classification accuracies for each digit in different defective CAs and the total classification accuracy. The total classification accuracy of D30 (LRS) CA was 44.5%, which are lower than that of D30 CA. These results reveal that the impact of degradation for the classification accuracy which is constructed with LRS stuck defects is more critical than HRS stuck defects. These results highlight that the impact of degradation on classification accuracy, when constructed with 'LRS stuck defects,' is more critical than that of 'HRS stuck defects.

Supplementary Information 7

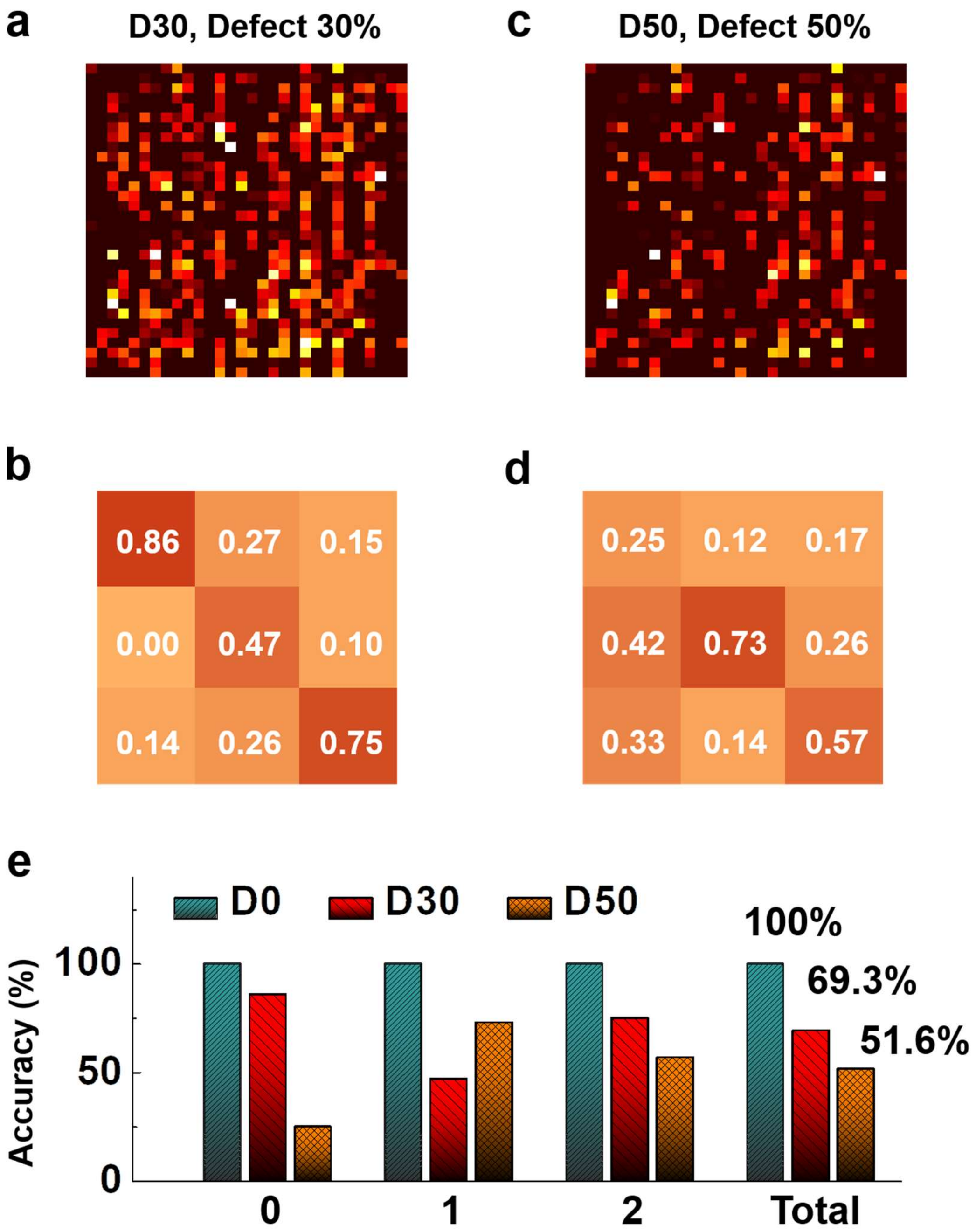

Supplementary Fig. S7: Hardware-based classification results using D30 and D50 in the condition of 50x read margin. **a** illustrates the conductance mapping for a defect rate of 30% (D30) of the CA, while **b** presents classification results for each digit. Similarly, **c** utilizing a defect rate of 50% (D50) for the CA, we have provided the conductance mapping result, and **d** the classification accuracy for each digit. e The overall classification accuracies are summarized.

The CA with defect rate D0 achieved a classification accuracy of 100%, while the CAs of D30 and D50 achieved classification accuracies of 69.3% and 51.6%, respectively. From this experiment, two main conclusions were drawn. Firstly, as demonstrated earlier in Fig 4 of the manuscript, the results showed a decrease in classification accuracy with increasing defect rate. Secondly, when compared to the results under the read margin of 15 conditions, an increase in accuracy of 0.8% and 1.8% was observed for D30 and D50 systems, respectively. Although there was an increase in accuracy, the magnitudes were not significant, and therefore, no significant differences based on the read margin could be observed.

Supplementary Note 1: The calculation process of energy efficiency of proposed 1 kb passive CA for implementing vector-matrix multiplication (VMM) operation.

To assess the energy efficiency of our proposed 1 kb passive Crossbar Array (CA) for the hardware-based implementation of Vector-Matrix Multiplication (VMM) operations, we calculated the 'Tera operations per second per Watt (TOPS/W)' using the equation:

$$\frac{TOPS}{W} = \frac{2\ \times N\ operations}{Read\ latency\ \times Power\ consumption}$$

In our study, we employed the parameters N=32, Read latency = 10 µs, and Power consumption = 1.47 µW, extracted from the defect-free (D0) CA, for our calculations. This yielded an energy efficiency of 4.35 TOPS/W for the VMM performance.

Reference

1. Jeon, K. et al. Self-rectifying resistive memory in passive crossbar arrays. Nat. Commu. **12(1)**. 2968 (2021).
2. Chen, J. et al. $LiSiO_x$-Based Analog Memristive Synapse for Neuromorphic Computing. IEEE Elec. Dev. Letters. **40**. 542-545.(2019)
3. Ryu, J. J et al. Highly Linear and Symmetric Weight Modification in HfO_2-Based Memristive Devices for High precision Weight Entries. Adv. Electron. Mater. **6.** 2000434. (2020).
